# Assays for studying normal *versus* suppressive ERAD-associated retrotranslocation pathways in yeast

**DOI:** 10.1016/j.xpro.2021.100640

**Published:** 2021-07-07

**Authors:** Satarupa Bhaduri, Sonya E. Neal

**Affiliations:** 1 Division of Biological Sciences, the Section of Cell and Developmental Biology, University of California San Diego, La Jolla, CA 92093, USA

**Keywords:** Cell Biology, Cell culture, Flow Cytometry/Mass Cytometry, Cell-based Assays, Cell separation/fractionation, Genetics, Model Organisms, Protein Biochemistry

## Abstract

In *S. cerevisiae*, we identified rhomboid pseudoprotease Dfm1 as the major mediator for removing or retrotranslocating misfolded membrane substrates from the ER (endoplasmic reticulum). Long-standing challenges with rapid suppression of *dfm1*-null cells have limited the biochemical study of Dfm1’s role in ER protein quality control. Here, we provide a protocol for the generation and handling of *dfm1*-null cells and procedures for studying normal vs. suppressive alternative retrotranslocation pathways. Our methods can be utilized to study other components involved in retrotranslocation.

For complete information on the generation and use of this protocol, please refer to Neal et al. (2017, 2018); Neal et al. (2019); Neal et al. (2020).

## Highlights

• Generation of *dfm1*-null yeast cells expressing a GFP-tagged ERAD-M substrate
• Detailed protocol on passaging *dfm1*-null cells to suppression
• Protocol of assays to analyze suppressed vs. non-suppressed ERAD pathways
• Analyze alternative ERAD retrotranslocation pathways when Dfm1 is absent

## Graphical abstract

**Figure:**
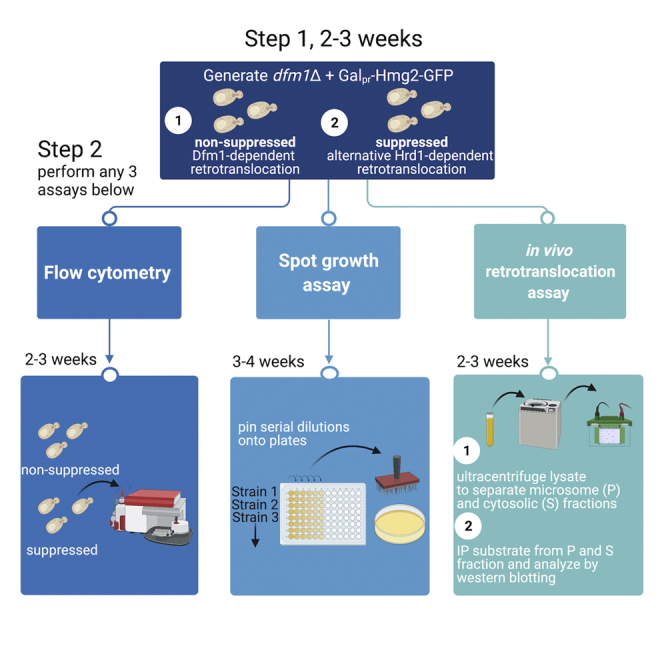

## Before you begin

You will need to generate *dfm1*Δ cells expressing GFP-tagged ERAD-M substrate, GALpr-HMG2-GFP that is driven by the GAL promotor. Briefly, *dfm1*Δ will be generated via PCR-mediated knockout followed by integration of GALpr-HMG2-GFP in *dfm1*Δ cells.

**Timing: 1 day**

Day 1:

1. Forward primer A and reverse primer B (Figure 1 and key resources table) were used to amplify the KanMx gene from plasmid pSN26 (key resources table), which encodes resistance to G418. The resulting product should contain the selectable marker flanked by 50 base pairs of the 5′ and 3′ regions, which are immediately adjacent to the coding region of the Dfm1 gene to be deleted. For the PCR reaction, we used Phusion polymerase (key resources table) for which the annealing step was performed at 59°C for 40 s followed by extension at 72°C for 1 min, 25–30 cycles.

**Figure 1:**
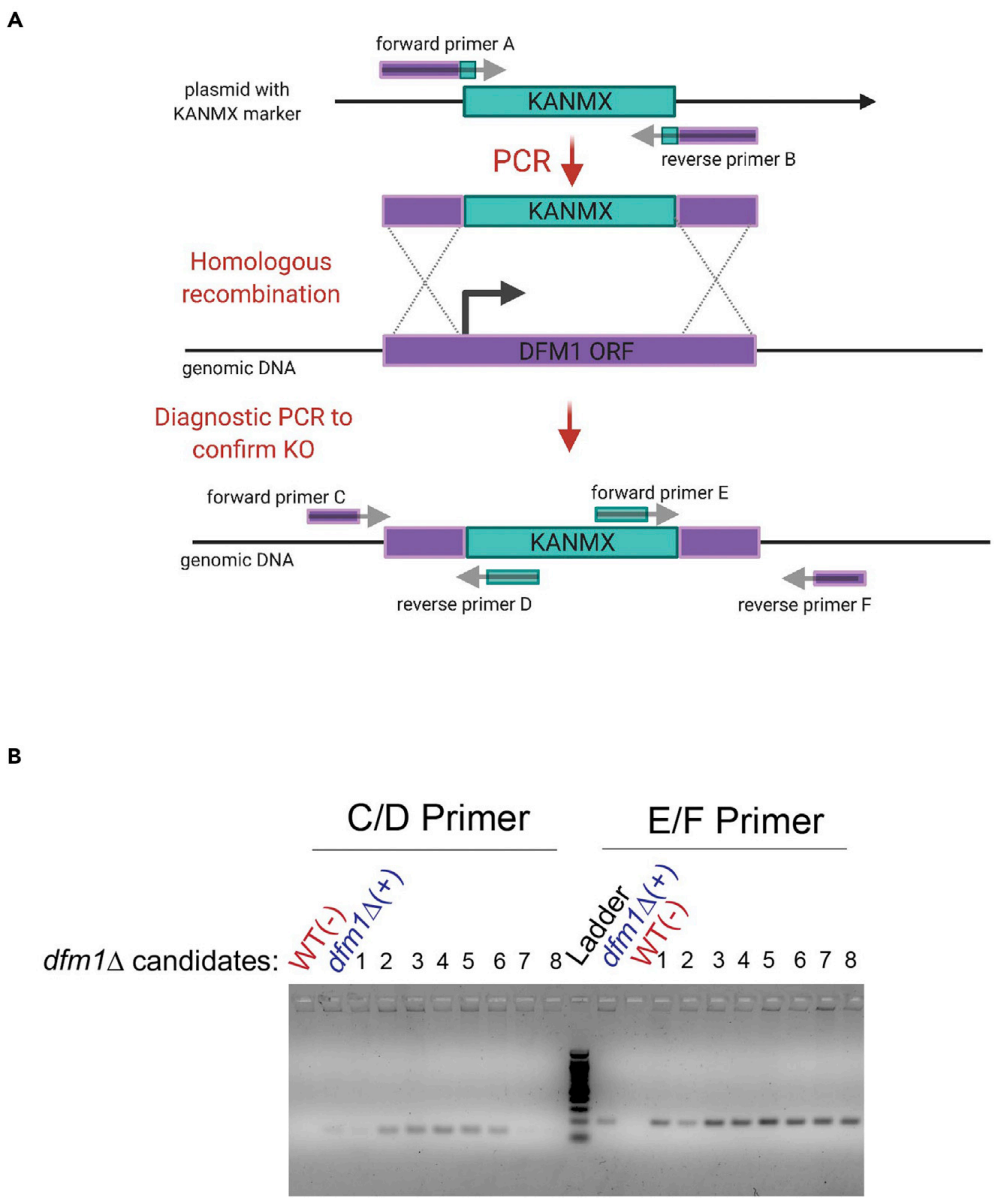
Schematic overview of the knock-out strategy by the one-step PCR method Primers are designed to contain “homologous arms” that are upstream and downstream of the Dfm1 ORF. (A) An antibiotic gene cassette (e.g., KanMx) is amplified by PCR and the PCR product is transformed to competent yeast cells. Homologous recombination will replace the targeted Dfm1 ORF with the KanMx cassette. (B) Representative gel image of PCR diagnostics on WT negative control strain, *dfm1*Δ positive control strain, and *dfm1*Δ candiates 1–8. Diagnostic with Primers C and D will yield ∼150 bp product whereas Primers E and F will yield ∼200 bp product.
2. Load amplified PCR product on 1% agarose gel and visualize PCR product (∼1.5 kb) using ChemiDoc.

**Pause point:** At this point, PCR reactions can be stored at −20°C for future use.

***Note:*** Dfm1 can be replaced with other markers such as NatMx and HphMx which encodes resistance to nourseothricin and hygromycin respectively. The primers used for KanMx-mediated knockout can be universally used to amplify NatMx and HphMx with plasmids pSN27 and pSN28, which encodes NatMx and HphMx respectively (see key resources table). Furthermore, other plasmids with appropriate markers can be utilized for knocking out Dfm1.

**CRITICAL:** PCR cleanup is not required, and 50 μL of PCR reaction can be used directly for each transformation (see below under “Generating dfm1Δ-null yeast strains”).

### Generating *dfm1*Δ-null yeast strains

**Timing: approximately 1 week**

Day 1:

3. To prepare yeast competent cells, inoculate a single colony of wildtype S288C cells into 5 mL of YPD medium and grow with rotation at 30°C for ∼16–20 h until culture reaches saturation with an OD_600_ (Optical Density at 600 nm) ∼3.0.

***Note:*** Other yeast strains such as W303, BY4741 and BY4742 can be also utilized for generating Dfm1 knockout.

Day 2:

4. Dilute saturated culture to 0.2 OD_600_ in total volume of 50 mL of YPD in a 250 mL Erlenmeyer flask and allow cells to double with shaking at 30°C for ∼3–4 h.
5. Once cells reach ∼0.6–0.9 OD_600_, pour 50 mL of culture into a 50 mL Falcon tube and pellet cells at 2,500 × g for 5 min at room temperature (20°C–25°C).
6. Discard supernatant and resuspend pellets directly in 5 mL of 1×TEL solution and rotate cells on a nutator overnight (16–20 h) at room temperature (20°C–25°C).

Day 3:

7. Spin cells at 2,500 × *g* for 5 min at room temperature (20°C–25°C).
8. Discard supernatant and resuspend pellet in 500 μL 1×TEL solution.

**Pause point:** At this point, competent cells remain in 1×TEL solution and can be used for transformation or stored in 4°C for ∼3 weeks for future use.

Day of transformation:

9. Mix 100 μL of yeast competent cells, 50 μg fish sperm DNA, and 50 μL of PCR reaction in a 1.5 mL Eppendorf tube. Incubate for 20 min at room temperature (20°C–25°C).
10. Add 700 μL of 40% PEG-TEL solution, vortex for couple seconds (setting on high) and incubate for 40 min at room temperature (20°C–25°C).

***Note:*** Important to include a no DNA control for yeast transformations so you have a sense for what the background growth is.

**CRITICAL:** Because 40% PEG-TEL is viscous, during the 40 min incubation with PEG-TEL, samples should be vortexed into solution every 10 minutes. An alternative method is to nutate the samples throughout the incubation period.

11. Heat shock samples in 42°C water bath for 7 min. Immediately after heat shock, spin sample briefly for 10 s (centrifuge setting: 10,000 × *g*). Discard supernatant and resuspend pellet in 50 μL of sterilized deionized water.
12. Add sterilized glass beads to YPD plates (∼10 beads per plate) followed by addition of cell suspension. Spread cells by shaking plates for several minutes. Pour out glass beads and incubate the plates at 30°C for 24 h.

Day 4:

13. At this point, there should be a thick lawn of cells grown on the YPD plate. If not, incubate the plate for additional time.
14. Replica plate cells onto YPD+G418 plates and incubate at 30°C for ∼3–4 days.

Day 8:

15. Once colonies are visible on plates, streak single colony transformants as wagon wheels on another YPD+G418 to ensure transformants breed true.

***Note:*** At this stage, we typically restreak ∼10–20 visible colonies.

16. For transformants breeding true, inoculate in 3 mL of YPD, rotate overnight (16–20 h) at 30°C and once cells reach saturation (OD_600_∼3.0), freeze all cultures immediately in 15% DMSO in 1.5 mL cryo-storage tubes at −80°C.
17. For confirmation of Dfm1 knockout, isolate genomic DNA from transformant as described in (Harju, Fedosyuk and Peterson, 2004). We typically obtain ∼200 ng/μL with a 260 nm/280 nm absorption of∼1.8 ,which indicates pure DNA. The genomic DNA is diluted to 10 ng/μL in deionized sterile water and 1 μL (10 ng) is used for PCR diagnostic amplification. Knockout was confirmed with forward primer C, reverse primer D, forward primer E and reverse primer F (Figures 1A and 1B & key resources table) that are outside of Dfm1 locus and within the KanMx gene.

***Note:*** For PCR amplification, it is also critical to include a negative control, wild-type genomic DNA. Furthermore, DFM1 knockout efficiency is ∼60%. Accordingly, by screening at least 10 colonies, we typically confirm knockout from ∼5–6 colonies.

**CRITICAL:** Because *dfm1Δ* cells have been shown to suppress, it is imperative that once transformants are obtained, that you freeze them (we usually freeze 10 transformants) and once validated, a representative set of 2–3 strains are transferred to the lab’s yeast storage collection. For freezing, prepare an overnight culture by inoculating 5 mL of YPD with a single colony of transformant and grow overnight (16–20 hours) with rotation at 30°C. Once cultures reach saturation growth (OD_600_∼3.0), add 850 μL of culture to 150 μL of DMSO (15%) in 1 mL cryo-storage tubes and place in −80°C freezer.

For additional advice for generating *dfm1Δ* strains, see troubleshooting section below.

### Integrating galactose-driven ERAD membrane substrate gene, Hmg2, into *dfm1*Δ-null yeast strains

**Timing: approximately 1–2 weeks**

***Note:*** For reference, check (Neal et al., 2020; Neal *et al.*, 2018; Flagg, Kao and Hampton, 2019) where Hmg2 was utilized.

Day 1:

18. Thaw out fresh *dfm1Δ*-null cells from −80°C freezer by plating on YPD plate. Incubate plates at 30°C for ∼3 days.
19. While *dfm1Δ* cells are growing, linearize a yeast integration plasmid pSN105(key resources table) containing GALpr-Hmg2-GFP by digesting 3 μg of plasmid with restriction digest enzyme (total volume 150 μL) that cuts once at the marker gene (e.g., StuI enzyme was used in our case for the ADE2 marker). Accordingly, the linearized plasmid should integrate at the *ade2-101* locus.
20. Check for linearization via 1% agarose gel and quench digestion reaction by incubating at 60°C for 20 min.

Day 4:

Prepare *dfm1Δ* competent cells by following steps 3–6 in major step above “Generating *dfm1*Δ-null yeast strains.”

Day 6:

21. Mix 100 μL of yeast competent cells, 50 μg fish sperm DNA, and 50 μL of digestion reaction containing 1 μg of linearized plasmid in a 1.5 mL Eppendorf tube. Incubate for 20 min at room temperature (20°C–25°C).
22. Add 700 μL of 40% PEG-TEL solution, vortex for couple seconds (setting on high) and incubate for 40 min at room temperature (20°C–25°C).
23. Heat shock samples by placing samples in a water bath set at 42°C for 7 min. Immediately after heat shock, spin sample briefly for 10 s (set at 10,000 × g). Discard supernatant and resuspend pellet in 50 μL of sterilized deionized water.
24. Add sterilized glass beads to SC-His plates (∼10 beads per plate) followed by addition of suspension cells. Spread cells by shaking plates for several minutes. Incubate the plates at 30°C for ∼2–3 days.

Day 9:

25. Once colonies are visible on plates, streak transformants as wagon wheels on another SC-His plate to ensure transformants breed true.

**CRITICAL:** Because *dfm1Δ* cells have been shown to suppress, it is imperative that once transformants are obtained, that they are frozen immediately (we usually freeze 10 transformants) and once validated, transfer a representative 2–3 strains to the lab yeast storage collection.

Check for integration by growth in media and 0.2% galactose induction of GALpr-Hmg2-GFP by flow cytometry (To use flow cytometry, see major step “Flow Cytometry to analyze for restored membrane substrate, Hmg2-GFP, degradation” below.

**Figure 2:**
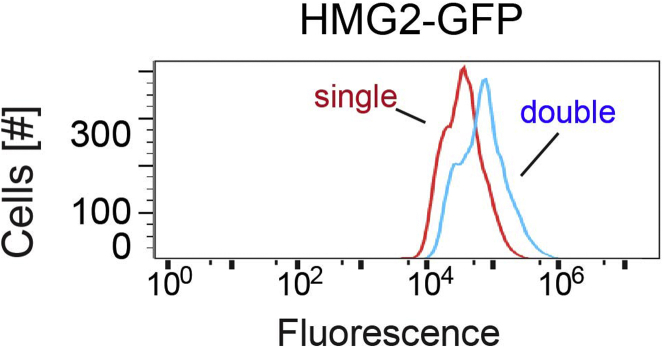
Mean fluorescence of single integrin *vs*. double integrin of Hmg2 Mean fluorescence of single-integrin of Hmg2 is ∼ ∼27 K (shown in red) whereas the mean fluorescence of double-integrin of Hmg2 is ∼ 58 K (shown in blue). Histograms of 10,000 cells are shown, with the number cells *versus* GFP fluorescence.

***Note:*** Because multiple integrations can occur in a single transformation, this step is critical for scanning transformants with a single integrant of GALpr-Hmg2-GFP (see Figure 2).

26. For transformants breeding true, inoculate in 3 mL of minimal media-His and freeze all cultures immediately with 15% DMSO in 1.5 mL cryo-storage tubes.

***Note:*** See Troubleshooting section below for common problems and solutions that arise in yeast transformation.

## Key resources table

**Table:** 

REAGENT or RESOURCE	SOURCE	IDENTIFIER
**Antibodies**		

Mouse monoclonal anti-GFP	Clontech Laboratories, Inc.	Cat#632381; RRID: AB_2313808
Mouse monoclonal anti-HA	Thermo Fisher Scientific	Cat#32–6700; RRID: AB_2533092
Rabbit polyclonal anti-myc	GenScript	Cat#A00172; RRID: AB_914457
Rabbit polyclonal anti-Cdc48	Neal et al., 2017	N/A
Mouse monoclonal anti-PGK	Thermo Fisher Scientific	Cat#459250; RRID: AB_2569747
Mouse monoclonal anti-Ubiquitin	Richard Gardner: University of Washington	N/A

**Bacterial and virus strains**		

*Escherichia coli* DH5 alpha Competent Cells	Thermo Fisher Scientific	Cat#18265017

**Chemicals, peptides, and recombinant proteins**		

MG132 (benzyloxycarbonyl-Leu-Leu-aldehyde)	Sigma-Aldrich	Cat# 474787; CAS: 133407-82-6
Tris-HCl	Fisher Scientific	Cat# BP153-500
NaCl	J.T. Baker	CAS: 7647-14-5
Glycine	Fisher Scientific	Cat# BP-381-500
20% SDS	Fisher Scientific	Cat#1311-200
Triton X-100 detergent	Fisher Scientific	Cat# BP-151-100
Sucrose	Fisher Scientific	Cat#S5-3
Methanol	Fisher Scientific	Cat#A412-500
EDTA	MP Biomedicals	CAS: 10378-22-0
Ultra-pure dithithreitol (DTT)	Invitrogen	Cat# P2325
Urea	Fisher Scientific	Cat#U15-500
Tween-20	Fisher Scientific	Cat#BP337-100
FBS	Thermo Fisher Scientific	Cat# 26140087
HCl	Fisher Scientific	Cat# A466-250
Sodium hydroxide (pellets)	Fisher Scientific	Cat# S318-500
Lithium acetate	Sigma	Cat# L4158
PEG 3350	Fisher Scientific	Cat# BP2331
Ethanol	Sigma	Cat# 459836
Sodium deoxycholate	Sigma	Cat# 30970
Raffinose	BD	CAS: 90000-940
Galactose	BD	CAS: 90000-926
Dextrose	BD	CAS: 8092678
MOPS	MP Biomedicals	Cat#102370
NEM	Sigma	Cat# SLBW6111
PMSF	Sigma	Cat# P7626
Benzamidine	Sigma	Cat# B6506
Leupeptin	Sigma	Cat# L2884
Pepstatin	Sigma	Cat# P5318
Caproic acid	Sigma	CAS: 60-32-2
(2-Aminoethyl)benzenesulfonyl fluoride hydrocholoride (AEBSF)	Sigma	Cat# A8456
Tosyl-phenylalanine chloromethyl-ketone (TPCK)	Sigma	Cat# T4376
L-Tryptophan	Sigma	CAS: 73-22-3
L-Adenine	MP Biomedicals	Cat# 100195
L-Leucine	MP Biomedicals	Cat# 4050512
L-Lysine	MP Biomedicals	Cat# 190224
L-Histidine	MP Biomedicals	Cat# 101954
L-Methionine	Sigma	CAS: 63-68-3
Na_2_HPO_4_	EMD Millipore	Cat# 3050917
Bromophenol Blue	Fisher Scientific	Cat# 175075
Bacto Peptone	BD	Cat# 8162527
Bacto Agar	BD	Cat# 8162556
Bacto Yeast Extract	BD	Cat# 8165971
DMSO	Fisher Chemical	Cat# 179119
6 mm Glass beads	Fisher Scientific	Cat# 11312D
Yeast Nitrogen Base	Sigma	Cat# Y0626
.5 mm Silica beads	BioSpec	Cat# MSPP-11079105Z
Cycloheximide	Sigma-Aldrich	Cat# C7698; CAS: 66–819
Protein A Sepharose	GE Healthcare	Cat# 17–0780-01
High Fidelity Phusion Polymerase	New England Biolabs	Cat# M0530L
PCR Clean-Up System	Promega	Cat# A9282
Ampicillin	BioPioneer	Cat# C0029
G418	BioPioneer	Cat# C0050
GFP-Trap Agarose	ChromoTek	Cat# gta-20
Tris base	Sigma	10708976001
KCl	Sigma	Cat# P3911
Dry milk	Fisher Scientific	Cat# NC9121673
Fish sperm solution, MB grade	Roche Diagnostics	Cat# 35954921

**Experimental models: organisms/strains**		

*Saccharomyces cerevisiae* BY4741	GE Dharmacon	Cat#YSC1048
*Saccharomyces cerevisiae* S288C	This study	N/A

**Oligonucleotides**		

Plasmid used		
pSN26 ARS/CEN KanMx/Ade2 markers		N/A
pSN105 GALpr-HMG2-GFP Yip HIS3/ADE2 markers		N/A
pSN27 ARS/CEN NatMx/Ade2 markers		N/A
pSN28 ARS/CEN HphMx/Ade2 markers		N/A
Primers used^1^		
Dfm1 KO with KanMX, PCR forward primer (A) **GTCAAATCAAAAACTATTTTCGAGGAAATA TGTTTAGCTTGCCTCGTCCC**	Eton Biosciences	N/A
Dfm1 KO with KanMx, PCR reverse primer (B) **GGCAAAGTACATAGAAATAGATAAAAGTTG TGGATGGCGGCGTTAGTATC**	Eton Biosciences	N/A
KO with KanMx diagnostic, PCR forward primer (C) **ACTTACCCGTTCGCGGCTCA**	Eton Biosciences	N/A
KO with KanMx diagnostic, PCR reverse primer (D) **GTACGGGCGACAGTCACATCA**	Eton Biosciences	N/A
KO with KanMx diagnostic, PCR forward primer (E) **CAGTTTCATTTGATGCTCGAT**	Eton Biosciences	N/A
KO with KanMx diagnostic, PCR reverse primer (F) **TTATCAATCGGTTGCTATGCC**	Eton Biosciences	N/A

**Software and algorithms**		

Prism 7 for Mac	GraphPad Software	https://www.graphpad.com/scientific-software/prism/
ImageJ	NIH	https://imagej.nih.gov/ij/
FlowJo	Neal et al., 2018	https://www.flowjo.com/solutions/flowjo
BD Accuri C6	BD Accuri	Cat # 653122

**Other**		

Microwave with at least 800 W power		N/A
Incubator at 30°C with air circulation		N/A
Shaker (at least 150 rpm) or rotating wheel		N/A
Milli-Q machine		N/A
Spectrophotometer for measuring yeast culture optical density (OD) at 600 nm		N/A
Flow cytometer (e.g., BD Accuri benchtop flow cytometer)		N/A
Electrophoresis power supply (e.g., Biorad PowerPac)		N/A
Wet transfer system (e.g., Bio-Rad Trans-blot Turbo Transfer System)		N/A
Mini-gel electrophoresis system (e.g., BioRad Mini-PROTEAN Tetra Cell)		N/A
Gel imager (e.g., Biorad ChemiDoc)		N/A

## Materials and equipment

The following reagents can be prepared ahead of time.

**Table:** 

Reagent	Final concentration
SDS, store at 20°C	20%
Tris-HCl pH 8, store at 20°C	100 mM
Na_2_HPO_4,_ pH 8, store at 20°C	100 mM
EDTA, store at 20°C	100 mM
Sorbitol, store at 20°C	2 M
NaCl, store at 20°C	2 M
Lithium Acetate, store at 20°C	1 M
Deoxycholate, store at 20°C	20%
PEG 3350, store at 20°C	50%
Urea, store at 20°C	10 M
MOPS pH 8, store at 20°C	1 M
FBS, store at −20°C	100%
CHX, store in ETOH at −20°C	50 mg/mL
MG132, store in DMSO at −20°C	25 mg/mL
NEM, make fresh	3.125 %
Raffinose, store at 20°C	20%
Galactose, store at 20°C	20%
Dextrose, store at 20°C	20%
PMSF make fresh at ETOH	1 mM
Benzamidine, store at DMSO at −20°C	5mM
Leupeptin, store at DMSO at −20°C	5 mg/mL
Pepstatin, store at DMSO at −20°C	5 mg/mL
Caproic acid, store DMSO at −20°C	6.25 mg/mL
AEBSF, store at DMSO at −20°C	6.25 mg/mL
TPCK, store at DMSO at −20°C	5 mg/mL
L-Tryptophan, sterile filter, and store at 4°C	100 × 4mg/mL)
L-Adenine, sterile filter and store at 20°C	100 × (2mg/mL)
L-Leucine, sterile filter and store at 20°C	250 × (15mg/mL)
L-Lysine, sterile filter and store at 20°C	500× (15 mg/mL)
L-Uracil, sterile filter and store at 20°C	100× (2 mg/mL)
L-Methionine, sterile filter and store at 20°C	500× (10 mg/mL)
G418, store at 4°C	500 mg/mL
Bromophenol Blue, dissolve 100 mg in 10 mL dd-H_2_O, store at 20°C	1%

***Note:*** Cycloheximide solution waste should be disposed by appropriate hazardous waste procedures.

***Note:*** G418 and Tryptophan shouldn’t be exposed to light upon storage.

***Note:*** Please refer to the product information for the shelf lives of the individual reagents listed here.

**Table:** Synthetic complete plates (SC-His plates)

Reagent	Final concentration	Amount
Yeast nitrogen base	n/a	14 g
Drop-out mix∗	n/a	4 g
20% Dextrose or Raffinose or Galactose	2%	100 mL
Agar	n/a	20 g
ddH_2_O		**up to 1000 mL**
∗Drop-out mix consists of all amino acids except for Histidine. Autoclave solution, pour plates and store in 4°C

**Table:** G418 plates for KanMx selection

Reagent	Final concentration	Amount
Yeast extract	n/a	10 g
Bacto-peptone	n/a	20 g
20% Dextrose	2%	100 mL
Agar	n/a	20 g
500 mg/mL G418	500 μg/mL	1 mL
ddH_2_O		**up to 1000 mL**
Autoclave solution, pour plates, store plates in 4°C

**Table:** Minimal Media -His

Reagent	Final concentration	Amount
Yeast nitrogen base	n/a	3.5 gr.
20% Raffinose or Dextrose	2%	50 mL
100× Tryptophan	1×	5 mL
100× Adenine	1×	5 mL
250× Leucine	1×	2 mL
500× Lysine	1×	1 mL
100× Uracil	1×	5 mL
500× Methionine	1×	1 mL
ddH_2_O		**up to 500 mL**
store in 20°C

**Table:** 5×TEL solution

Reagent	Final concentration	Amount
1 M Lithium acetate	50 mM	25 mL
1 M Tris-HCl pH 8	50 mM	25 mL
100 mM EDTA	1 mM	5 mL
ddH_2_O	n/a	445 mL
**Total**		**500 mL**
Sterile filter before use, store in 20°C

**Table:** 40% PEG-TEL solution

Reagent	Final concentration	Amount
5×TEL solution	1×	10 mL
50% 3350 PEG	40%	40mL
**Total**		**50 mL**
Sterile filter before use, store in 20°C

**Table:** MF Buffer

Reagent	Final concentration	Amount
2 M Sorbitol	300 mM	15 mL
2 M NaCl	100 mM	5 mL
100 mM Tris-HCl pH 8	20 mM	20 mL
ddH_2_O	n/a	60 mL
**Total**		**100 mL**
store in 20°C

**Table:** SUME Buffer

Reagent	Final concentration	Amount
20 % SDS	1%	5 mL
10 M Urea	8 M	80 mL
1 M MOPS pH 8	10 mM	1 mL
100 mM EDTA	10 mM	10 mL
ddH_2_O	n/a	4 mL
**Total**		**100 mL**
store in 20°C

**Table:** Immunoprecipitation Buffer:

Reagent	Final concentration	Amount
100 mM Na_2_HPO_4,_ pH 8	15 mM	75 mL
2 M NaCl	150 mM	37.5 mL
1 M MOPS pH 8	10 mM	5 mL
Triton X-100	2%	10 mL
20% SDS	0.1%	2.5 mL
10 % Deoxycholate	0.5%	25 mL
100 mM EDTA	10 mM	50 mL
ddH_2_O	n/a	295 mL
**Total**		**500 mL**
store in 20°C

**Table:** Immunoprecipitation Wash Buffer:

Reagent	Final concentration	Amount
100 mM Na_2_HPO_4,_ pH 8	50 mM	250 mL
2 M NaCl	10 mM	2.5 mL
ddH_2_O	n/a	247.5 mL
**Total**		**500 mL**
store in 20°C

**Table:** 2×USB SDS-PAGE loading buffer:

Reagent	Final concentration	Amount
1 M MOPS	75 mM	3.75 mL
20% SDS	4%	10 mL
Dithiotreitol (DTT)	1 mM	1.53 g
Urea	8 M	24 g
1 % Bromophenol Blue		1 mL
ddH_2_O		**up to 50 mL**
store in −20°C

**Table:** 8×SDS-PAGE Buffer

Reagent	Final concentration	Amount
Tris Base	400 mM	24.24 g
Glycine	3.1 M	115.2 g
20% SDS	1.6%	40 mL
ddH_2_O		**Up to 500 mL**
Adjust pH to 7.5 using HCl, store in 20°C

**Table:** 20×TBHST solution

Reagent	Final concentration	Amount
NaCl	2.13 M	80 g
KCl	54 mM	2 g
Tris Base	33 mM	30 g
Tween-20	9%	45 mL
ddH_2_O		**Up to 500 mL**
store in 4°C

**Table:** 2% milk solution

Reagent	Final concentration	Amount
Dry milk	2%	1 g
1×TBHST	n/a	up to 50 mL
**Total**		**50 mL**
store in 4°C

**Table:** 5% milk solution

Reagent	Final concentration	Amount
Dry milk	5%	2.5 g
1×TBHST	n/a	up to 50 mL
**Total**		**50 mL**
store in 4°C

**Table:** 10% FBS solution

Reagent	Final concentration	Amount
100% FBS	20%	10 mL
1×TBHST	n/a	up to 50 mL
**Total**		**50 mL**
Aliquot FBS in sterile conditions, store in 4°C		

## Step-by-step method details

### Culturing and passaging *dfm1*Δ-null +GAL_pr_-Hmg2-GFP cells to suppression

**Timing: [2–3 weeks]**

**Figure 3:**
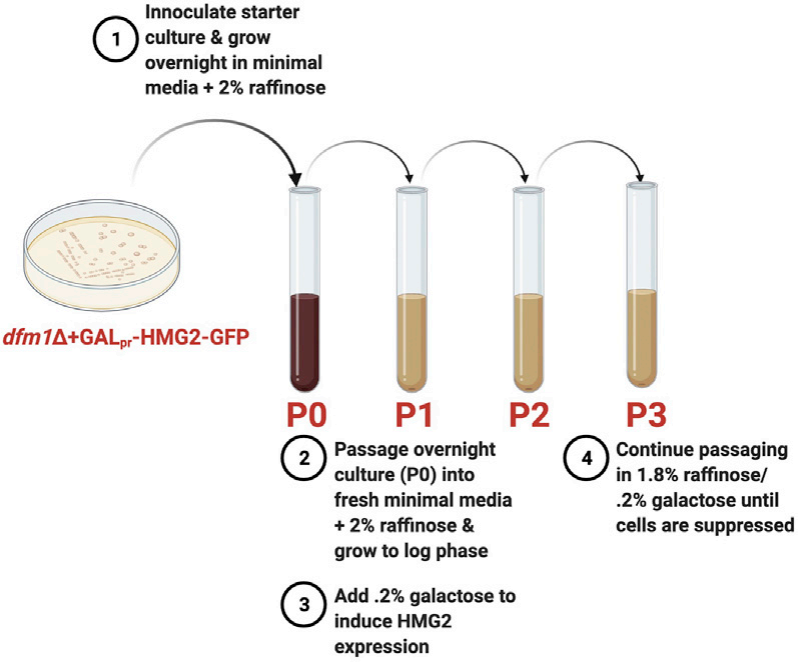
Schematic of passaging *dfm1*Δ+GAL_pr_-Hmg-GFP cells to suppression

This section outlines how *dfm1*Δ-null yeast cells containing GAL_pr_-Hmg2-GFP are passaged into fresh minimal media overtime to generate completely suppressed cells with restored ERAD-M retrotranslocation (Figure 3).

Day 1

1. Thaw *dfm1*Δ-null yeast cells containing GAL_pr_-Hmg2-GFP from freezer by streaking cells directly on SC-His plates.
2. Incubate SC-His plate in 30°C incubator for ∼3 days.

Day 4

3. Prepare starter culture by inoculating a single colony into 3 mL of minimal media-His supplemented with 2% raffinose.

**CRITICAL:** Raffinose is used instead of dextrose to avoid repression of the GAL promoter.

4. Grow starter culture overnight (16–20 h) until cells are grown to saturation (OD_600_>1).

***Note:****dfm1*Δ-null cells grow slower than wildtype cells. Wildtype growth rates typically have 90 min doubling time in glucose, 180 min doubling time in raffinose, and 150 min doubling time in galactose whereas *dfm1*Δ-null growth rates have 120 min doubling time in glucose, 240 min doubling time in raffinose, and 200 min doubling time in galactose.

Day 5

5. Dilute cells to OD_600_ ∼0.1 in total volume of 3 mL of minimal media -His supplemented with 2% raffinose and incubate with rotation at 30°C for 4–5 h; allowing cells to double or grow to an early log-phase (OD_600_ ∼0.2–0.3). This culture is designated as Passage 0 **(P0).**
6. For strong expression of Hmg2-GFP under the control of the galactose-inducible promoter, once cells have grown to early log phase (OD_600_∼.3-.8), induce cells with the addition of galactose at a final concentration of 0.2% (v/v).
7. Incubate at 30°C with rotation for ∼24 h or until cells are grown to saturation (OD_600_>1)

Day 6

8. Dilute cells to OD_600_ ∼0.1 in total volume of 3 mL of minimal media -His supplemented with 1.8% raffinose/0.2% galactose and incubate at 30°C with rotation for 24 h or until cells are grown to saturation (OD_600_>1). Designate this new culture as (**P1).**

Day 6–21

Continue passaging until *dfm1*Δ-null cells are completely suppressed (see major step below “Flow Cytometry to analyze for restored membrane substrate, Hmg2-GFP, degradation” for details on how to analyze cells for suppression). We typically see complete suppression by **P10**.

**CRITICAL:** It is important to passage cells immediately after culture reach saturation phase. Sustained growth in saturation phase makes it difficult for cells to recover after dilution. Refer to *troubleshooting section* below.

**CRITICAL:** For growth, minimal medium is used rather than YPD to reduce background fluorescence which will not interfere with flow cytometry fluorescent readouts.

### Flow cytometry to analyze for restored membrane substrate, Hmg2-GFP, degradation

**Timing: [2–3 weeks]**

This section outlines how Hmg2-GFP steady-state levels can be measured by flow cytometry throughout different passaging stages of *dfm1*Δ-null cells.

Controls and samples used:

• Non-induced control: cells are passaged continuously in the presence of glucose
• Degraded Hmg2 control: WT strains + GAL_pr_-Hmg2-GFP are passaged in induced galactose condition. This is a control for what steady-state Hmg2 levels would be when undergoing ERAD degradation.
• Stabilized Hmg2 control: *cdc48-2* + GAL_pr_-Hmg2-GFP passaged in induced galactose condition. This is a retrotranslocation-deficient control for what stabilized steady-state levels of Hmg2 would be.
• Sample 1: P0 *dfm1*Δ + GAL_pr_-Hmg2-GFP is non-passaged and non-suppressed sample, which should look like control #3.
• Sample 2: passaged *dfm1*Δ + GAL_pr_-Hmg2-GFP is suppressed strain, which should look like control #2.

Biological triplicates were used for each strain and flow cytometry analysis was performed as technical triplicates.

Day 1

**Figure 4:**
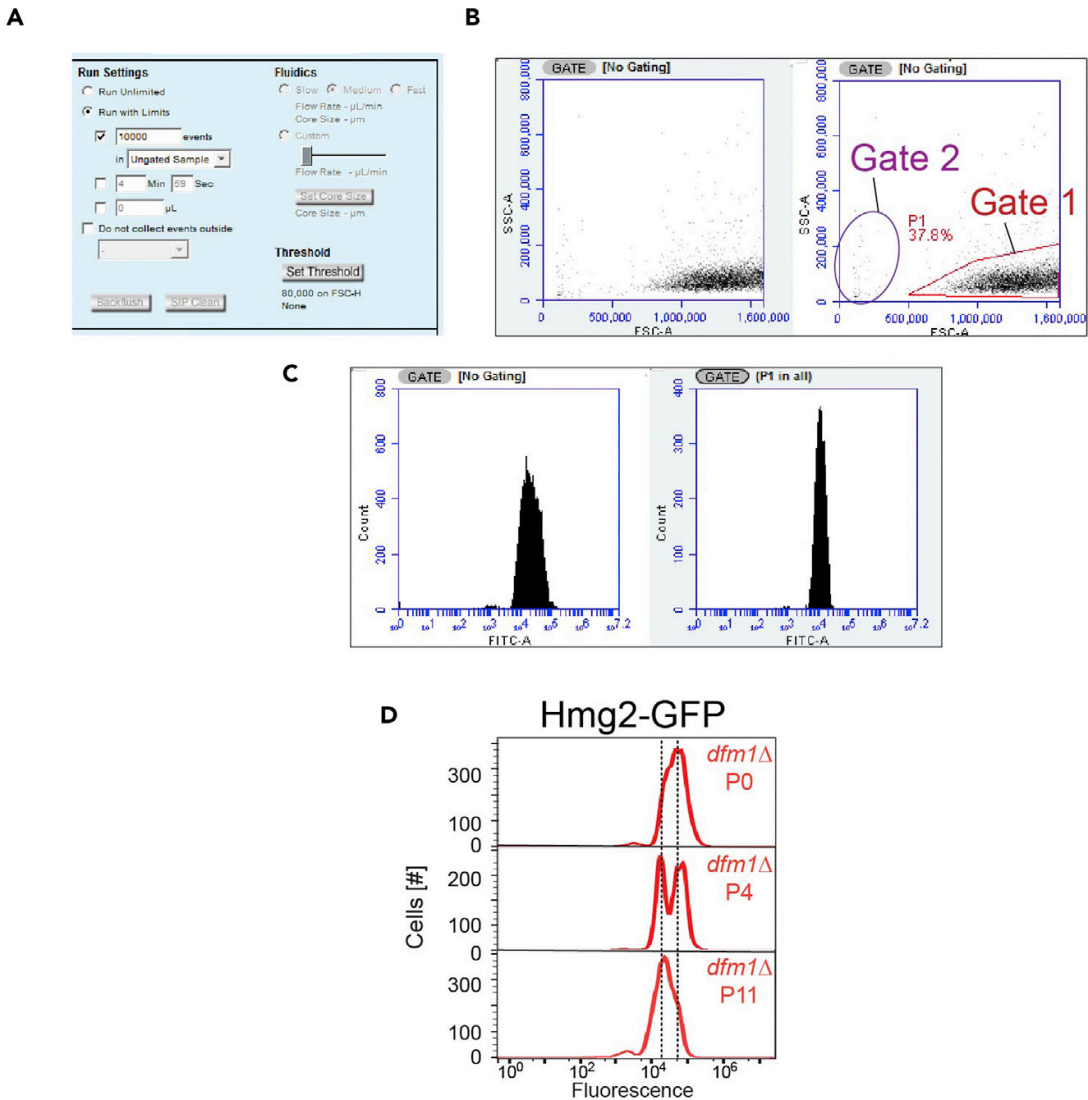
Selecting flow cytometry gates to determine mean fluorescence of cell population (A) Different parameter settings on BD Accuri flow cytometer before samples are analyzed. Run Settings: Run with Limits at 10,000 events. Fluidics: Medium flow rate for yeast. (B) Density plot displaying all events detected by flow cytometry. Gate 1 (in red) is drawn to isolate intact yeast cells, which is used for generating a histogram plot for mean fluorescence. Gate 2 (in purple) represents ruptured or damaged cells. (C) Entire cell population and Gate 1 cell population were used to generate histograms with the number of cells versus GFP fluorescence (FITC-A). (D)Analysis of nonsuppressed *dfm1*Δ cells (P0), passaged *dfm1*Δ cells (P4) and suppressed passaged *dfm1*Δ cells (P11) by flow cytometry. Mean fluorescence for P0 cells are ∼50K whereas suppressed P11 cells are ∼20 K. Histograms of 10,000 cells are shown, with the number cells *versus* GFP fluorescence.

9. Thaw, inoculate, and passage *dfm1*Δ-null yeast cells containing GALpr-Hmg2-GFP as indicated above.
10. For P0 analysis of Hmg2-GFP levels: Once *dfm1*Δ-null yeast cells containing GALpr-Hmg2-GFP have been induced with galactose for at least two hours and grown to OD_600_∼0.4, take 300 μL of P0 cells and analyze for mean fluorescence levels using BD Biosciences FACS Calibur flow cytometer.
11. Adjust the following settings before analyzing samples on flow cytometer (Figure 4A):

**Run Settings:** *Run with limits, 10,000 events*

**Fluidics flow rate:** *Medium*

12. Run samples by aliquoting 300 μL of suspended cells into sample tubes and placing the tube on sample collector.
13. **Data acquisition:** For each run, create two plots: density (side scatter SSC vs. forward scatter FSC) and histogram plots (Cell Count vs. 530 filter for GFP FITC-A).
14. Draw a gate around the population of intact yeast cells and display gated population as a histogram (Cell count vs. 530 filter for GFP FITC-A) to obtain the mean fluorescence of the gated population (Figures 4B and 4C).
15. For analysis of other passages, once cells are passaged and diluted in fresh minimal media, allow cells to double to OD_600_∼.4 and analyze for mean fluorescence via flow cytometer.

**CRITICAL:** Analyzing cells in saturated growth phase will yield large cellular debris and an abnormal distribution of fluorescence. Accordingly, it is important to analyze cells in the log-phase. Finally, all flow cytometry readings were performed directly from minimal media since it has negligible background fluorescence.

### Spot growth assay of non-suppressed and suppressed *dfm1*Δ-null cells

**Timing: [3–4 weeks]**

**Figure 5:**
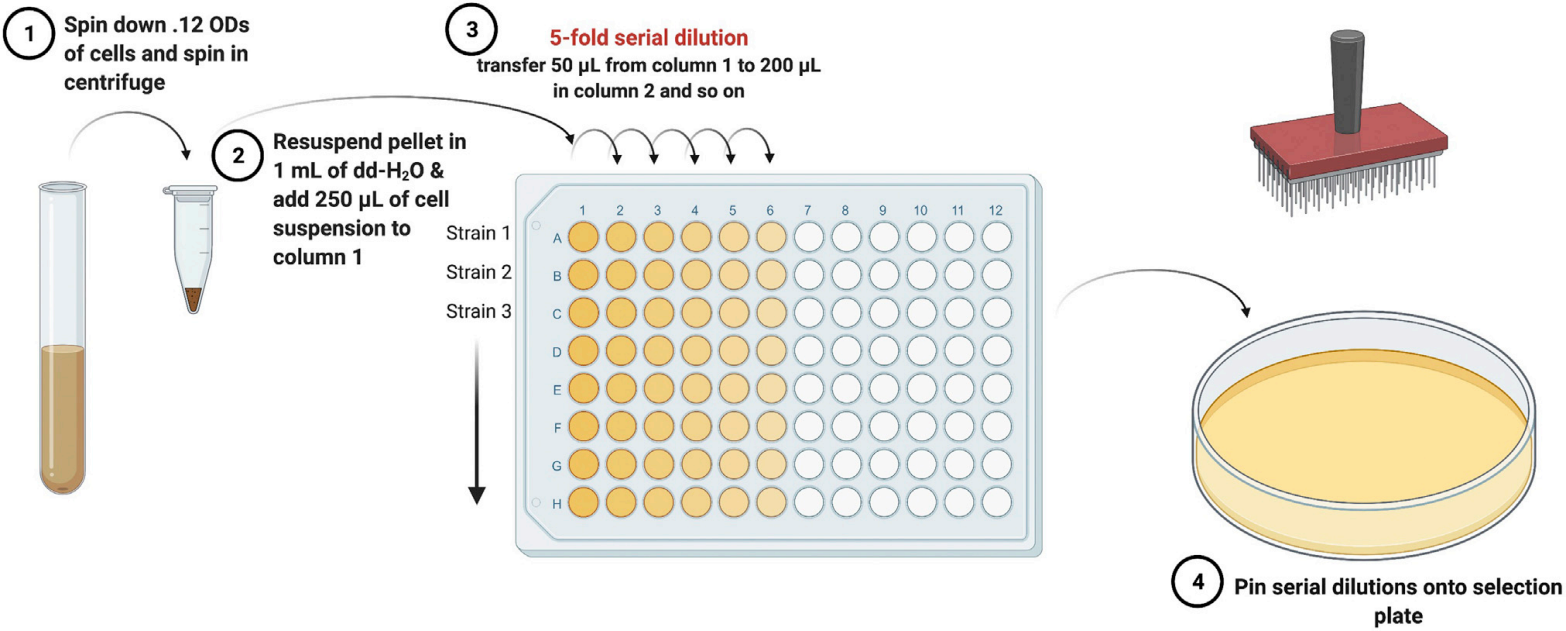
Schematic of spot assay used to analyze suppressed *versus* non-suppressed *dfm1*Δ+GAL_pr_-HMG-GFP

This section outlines how spot growth assay can be utilized to demonstrate normal growth for suppressed *dfm1*Δ-null cells vs. a growth defect in non-suppressed *dfm1*Δ -null cells (Figure 5).

Controls used

Negative control: WT and *cdc48-2* strains expressing both GAL_pr_-Hmg2-GFP and empty vector.
Negative control: *dfm1*Δ expressing empty vector.

Biological triplicates were used for each strain and the growth assay was performed as technical triplicates.

Day 1

16. Thaw *dfm1*Δ-null yeast cells containing GALpr-Hmg2-GFP from freezer by streaking on SC-His plates.
17. Incubate SC-His plate in 30°C incubator for ∼3 days.

Day 4

18. **For growth analysis of P0 cells:** Inoculate a single colony into 3 mL of minimal media supplemented with 2% raffinose. This culture is designated as Passage 0 (Passage 0).
19. Incubate at 30°C with rotation overnight (16–20 h) until cells are grown to saturation (OD_600_>1).

Day 5

20. Dilute cells to OD_600_ ∼0.1 and incubate with rotation at 30°C for 4–5 h; allowing cells to double or grow to an early log-phase with OD_600_ ∼0.2–0.3.

Day 6

21. Pellet 0.12 OD of cells by spinning at 14,000 × *g* for 2 min at room temperature and resuspending pellets in 1 mL of sterilized dH_2_O.
22. Transfer 250 μL of each sample to a 96-well plate and perform a five-fold serial dilution in dH_2_O of each sample to obtain a gradient of 0.03–0.0000096 OD cells (Figure 5). 8. Pin the cells using the 8×12 pinning apparatus onto synthetic complete (-His) agar plates supplemented with 2% dextrose or 2% galactose.
23. Air-dry the pinned droplets of cells under the flame in sterile conditions, seal the plates with parafilm and incubate at 30°C.

***Note:*** To make the droplets absorb faster and avoid droplet from running along the plate, it is important to pre-dry the plates with lids off in the Biosafety Cabinet or under a flame before spotting.

**Figure 6:**
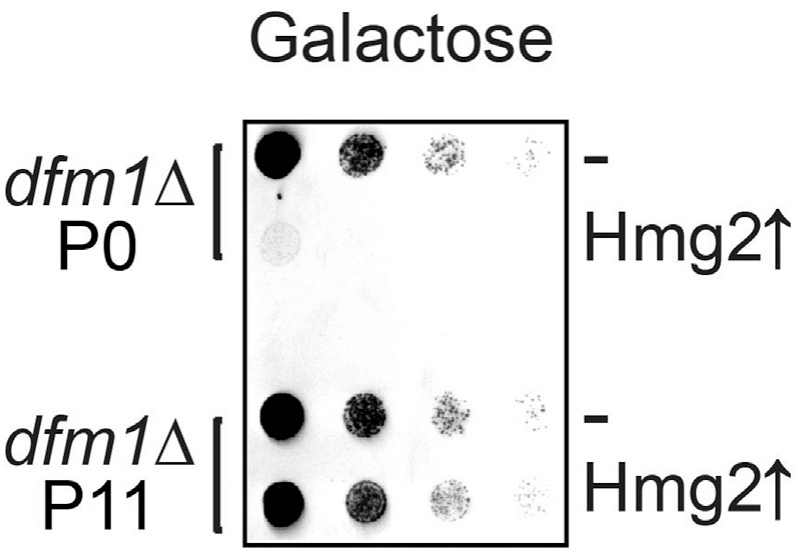
Strongly expressed integral membrane substrates cause a growth defect in P0 non-suppressed *dfm1*Δ cells and restored growth in P11 suppressed *dfm1*Δ cells Non-passaged *dfm1*Δ cells (P0) or cells passaged to suppression (P11) were assessed for growth defect in the dilution assay by spotting 5-fold dilutions of cells on galactose-containing plates to drive Hmg2-GFP overexpression, and plates were incubated at 30°C.

24. Remove the plates from the incubator for imaging with the ChemiDoc Imager (**Setting:** UV-Trans) or a camera as an alternative on Day 3 and 7.
25. **For growth analysis of suppressed passaged cells:** Once cells have been validated for suppression by the flow cytometer, pellet 0.12 OD of cells and follow steps 5–10 (Figure 6).

### *In vivo* retrotranslocation assay of suppressed and non-suppressed *dfm1*Δ-null cells

**Timing: [2–3 weeks]**

**Figure 7:**
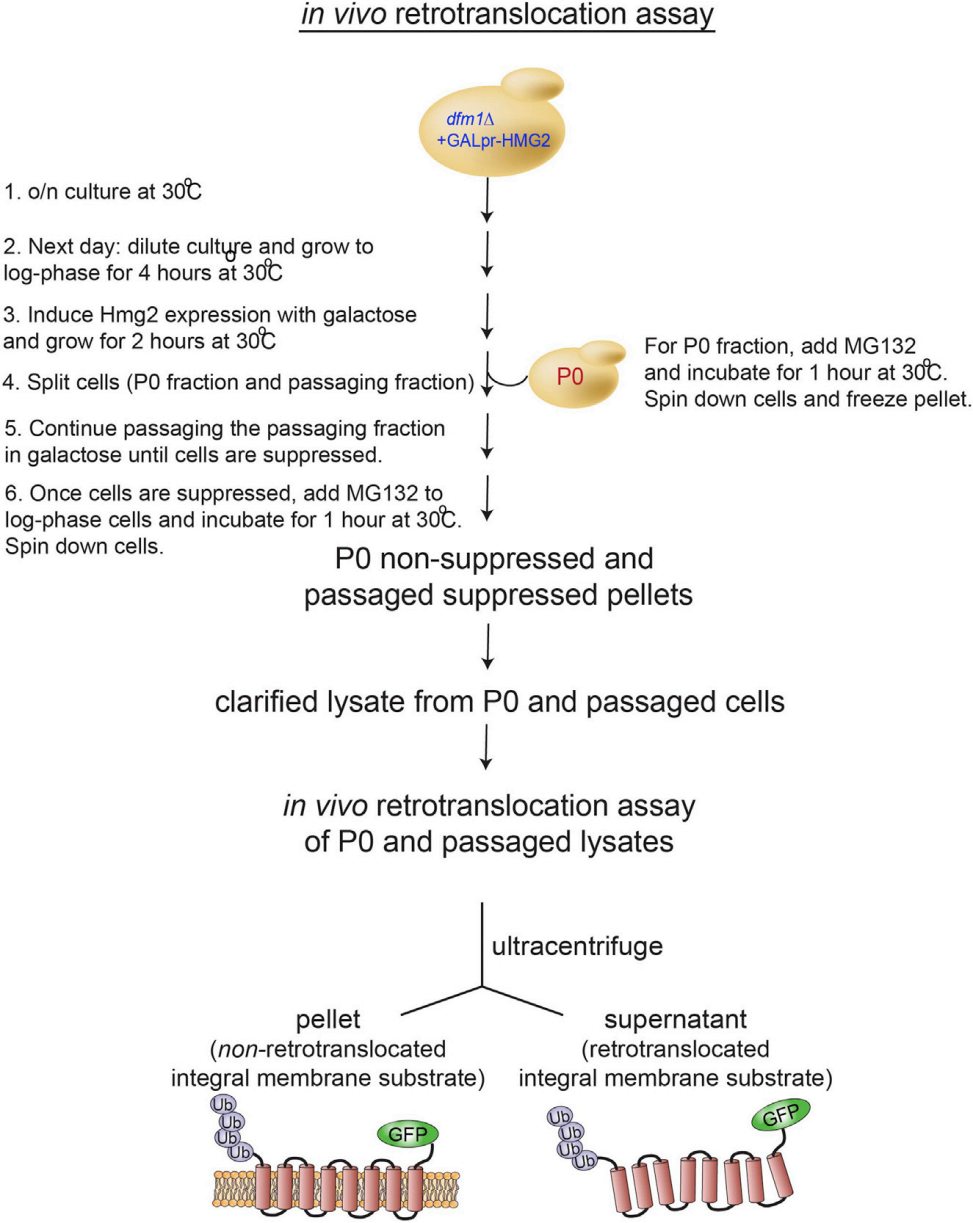
Depiction of *in vivo* retrotranslocation assay for analyzing membrane-bound and retrotranslocated membrane substrate, Hmg2-GFP Adapted from (Neal et al., 2019).

This section outlines how the *in vivo* retrotranslocation assay can demonstrate membrane substrate retrotranslocation deficiency for non-suppressed *dfm1*Δ -null cells vs. restored retrotranslocation in suppressed *dfm1*Δ-null cells (Figure 7).

Controls and Samples used:

• Degraded Hmg2 control: WT+ GAL_pr_-Hmg2-GFP for retrotranslocation.
• Stabilized Hmg2 control: *cdc48-2*+ GAL_pr_-Hmg2-GFP for being retrotranslocation-deficient.
• Sample 1: P0 *dfm1*Δ + GAL_pr_-Hmg2-GFP is non-passaged and non-suppressed sample, which should look like control #2.
• Sample 2: passaged *dfm1*Δ + GAL_pr_-Hmg2-GFP is suppressed strain, which should look like control #1.

Biological replicates were used for each strain and the *in vivo* assay was performed as technical triplicates.

Day 1:

26. Thaw *dfm1*Δ-null yeast cells containing GALpr-Hmg2-GFP from freezer by streaking directly on SC-His plates.
27. Incubate SC-His plate in 30°C incubator for ∼3 days.

Day 4:

28. Inoculate a single colony into 10 mL of minimal media supplemented with 2% raffinose. This culture is designated as Passage 0 (Passage 0).
29. Incubate at 30°C with rotation overnight (16–20 h) until cells are grown to saturation (OD_600_>1).

Day 5:

30. Dilute cells to OD_600_ ∼0.1 in fresh minimal media supplemented with 2% raffinose (total volume= 50 mL in 250 mL Erlenmeyer flask) and incubate with shaking at 30°C for 4 h; allowing cells to double or grow to an early log-phase (OD_600_ ∼0.2–0.3).
31. Once cells have grown to early-log phase (OD_600_∼0.2–0.3), remove 3 mL of cells suspension and add to culture tube. Continue passaging cells as outlined in major step above “Culturing and passaging *dfm1*Δ-null +GAL_pr_-Hmg2-GFP cells to suppression” (Steps 6–8) until cells are completely suppressed.
32. **Non-suppressed, non-passaged cells:** For the rest of the culture (∼47 mL), add MG132 at a final concentration of 25 μg/mL and incubate for 1 h with shaking for 2 h.

***Note:*** MG132 is a proteasome inhibitor that allows for accumulation of retrotranslocated HMG2 in cytosol and easier detection on western blot. We typically obtain efficient inhibition by MG132 with 1–2 hours of incubation.

33. After incubation with MG132 pellet 15 OD of cells in 50 mL falcon tubes by centrifuging at 1,000 × *g* for 5 min in room temperature.
34. Discard supernatant and resuspend the pellets in sterile deionized water and centrifuge at 1,000 × *g* for 5 min in room temperature.

**Pause point:** At this point, cells can be stored in −80°C freezer and for future use until suppressed passaged cells are ready for analysis in the retrotranslocation assay.

35. **Passaged suppressed cells:** Once cells are suppressed through continued galactose induction during passaging, dilute cells to OD_600_ ∼0.1 in fresh minimal media supplemented with 1.8% raffinose/ 0.2% galactose (total volume= 50 mL in 250 mL Erlenmeyer flask) and incubate with shaking at 30°C for 4 h; allowing cells to double or grow to an early log-phase (OD_600_ ∼0.2–0.3). Follow steps 43–45.
36. Resuspend passaged and non-passaged cell pellets in 400 μL of sterile deionized water and prepared for bead lysis by dividing samples into 4×2 mL Eppendorf tubes (∼100 μL of cell suspension per tube). Spin tubes at 10,000 × *g* rpm for 2 min at room temperature and aspirate supernatant.
37. Resuspend each pellet with 100 μL of MF buffer supplemented with the following protease inhibitors 1 mM PMSF, 260 μM AEBSF, 100 μM leupeptin, 76 μM pepstatin, 5 mM aminocaproic acid, 5 mM benzamidine, and 142 μM TPCK.

***Note:*** phenylmethylsulphonyl fluoride (PMSF) is prepared fresh for each experiment.

38. Add 0.5 mM silicone beads to meniscus of cell suspension. Vortex samples on multi-vortexer set at top speed for 6 × 1-min intervals with 10 min intervals on ice between each vortexing.
39. Check cells under the microscope (20 × magnification) for lysis efficiency.

***Note:*** Lysed cells are distinguishable by their fragmented shape and for optimal yield it is critical to achieve ∼80–90% lysis efficiency. See troubleshooting section below for more details on lysing.

**CRITICAL:** At the point, samples and all solutions should be kept on ice.

40. Add 100 μL of chilled MF buffer with PIs to each eppendorf tube and combine lysates by transferring to a new 1.5 mL Eppendorf tube with a 1 mL pipette. Centrifuge at 2,500 × *g* for 5 min at 4°C to remove cell debris.
41. Transfer clarified supernatant to ultracentrifugation tube.
42. Ultracentrifuge the clarified lysate at 100,000 × *g* for 15 min at 4°C to separate the pellet microsome fraction **(P)** and cytosolic supernatant fraction **(S).**
43. Resuspend pellet in 200 μL SUME buffer with PIs and NEM.
44. Add 600 μL of IPB with PIs and NEM to the **(S)** fraction and resuspended **(P)** fraction.
45. For immunoprecipitation of Hmg2-GFP add 15 μL of rabbit polyclonal anti-GFP antisera to the **(P)** and **(S)** samples.
46. Incubate the samples on ice for 5 min, spin at 14,000 × *g* for 5 min, and remove the supernatant to a new 1.5 mL eppendorf tube and incubate overnight with gentle mixing using a nutator at 4°C.

***Note:*** For equilibrating Protein A-Sepharose, do this before you begin the retrotranslocation assay:

Add Protein-A Sepharose to 50 mL Falcon tube. Fill tubes with ∼40 mL of deionized water and place tube on ice. Once beads settle to the bottom, carefully pour out water (it is ok to leave residual water). Repeat these 6 times with deionized water. After final rinse with water, add 6 mL of IPB. This solution is suitable for storage at 4°C for future use.

47. Add 100 μL of equilibrated Protein A-Sepharose to the samples and incubate for 2 h at 4°C with gentle mixing using a nutator.
48. Wash Protein A-Sepharose beads twice by adding 900 μL of IPB followed by brief spin of 1,000 × *g* for 30 s at room temperature and aspirating supernatant with an 18-gauge syringe needle. Repeat again with addition of IPB to beads.
49. Wash beads once more by adding 900 μL of IPW followed by brief spin of 1,000 × *g* for 30 s at room temperature and aspirating beads to dryness using a 30-gauge syringe needle.
50. Resuspend beads in 60 μL of 2× urea sample buffer and solubilize samples by incubating at 55°C for 10 min.
51. Spin samples at 14,000 × *g* for 5 min at room temperature. The eluted proteins are removed to a new tube.
52. Eluted proteins are resolved by SDS-PAGE using 8% gels, transferred to nitrocellulose membrane by electroblotting at 15 mAmp for 15 min using a TransBlot.
53. Immunoblot with monoclonal anti-ubiquitin (1:4,000 dilution) anti-GFP (1:10,000 dilution) along with Goat anti-mouse (Jackson ImmunoResearch, West Grove, PA) conjugated with horseradish peroxidase (HRP) recognized the primary antibodies. Immunoblotting was carried as described in (Neal et al., 2019).

## Expected outcomes

Under “Flow Cytometry to analyze for restored membrane substrate, Hmg2-GFP, degradation,” Hmg2-GFP levels range from being stabilized to being degraded as *dfm1*-null cells are being suppressed. For example, P0 cells should have stabilized Hmg2-GFP levels with a mean fluorescence of ∼50K. P6 cells should have mixed population of cells with mean fluorescence of ∼50K and ∼20K. Finally, P10 cells and on should have majority of cells suppressed in which Hmg2-GFP levels are restored to degradation levels of ∼20K (Figure 4D).

Under “Spot growth assay of non-suppressed and suppressed *dfm1*Δ-null cells,” no growth defect should be observed in passaged suppressed *dfm1*Δ cells (P11) in comparison to non-passaged non-suppressed *dfm1*Δ cells (P0) (Figure 6) demonstrating that suppressed *dfm1*Δ null strains with alleviated retrotranslocation function have normal growth fitness.

**Figure 8:**
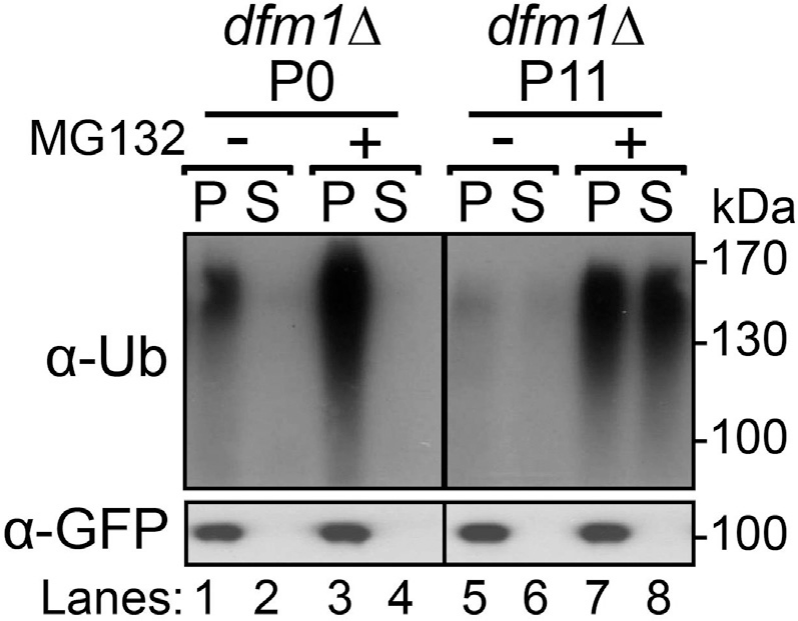
*In vivo* Hmg2-GFP retrotranslocation completely restored *dfm1*Δ suppressed cells Crude lysate was prepared from the indicated strains treated with vehicle or MG132 (25 μg/mL). Lysates were ultracentrifuged to discern ubiquitinated Hmg2-GFP that either has been retrotranslocated into the soluble fraction (S) or remained in the membrane (P). Following fractionation, Hmg2-GFP was immunoprecipitated from both fractions, resolved on 8% SDS-PAGE and immunoblotted with α-GFP and α-Ubi. Adapted from (Neal et al., 2018).

Under “*In vivo* retrotranslocation assay of suppressed and non-suppressed *dfm1*Δ-null cells,” non-passaged *dfm1Δ* P0 shows the typical buildup of ubiquitinated Hmg2-GFP in the pellet fraction in both untreated and MG132 treated cells (Figure 8). In striking contrast, suppressed P11 *dfm1Δ* cells shows normal Hmg2-GFP retrotranslocation, with buildup of ubiquitinated Hmg2-GFP is observed in both the pellet and supernatant fraction in MG132 treated cells.

## Limitations

*dfm1Δ*-null strains are susceptible to suppression. Many factors that can trigger and influence the rate of suppression included multiple rounds of yeast transformations, growth at high temperatures and strong expression of ERAD membrane substrates. As such, experiments requiring use of temperature-sensitive mutants or involving multiple rounds of transformations will trigger suppression and will pose a challenge in studying Dfm1’s contributions to ERAD. Below we outline tips, focused on handling *dfm1*-null strains.

## Troubleshooting

### Problem 1

Homologous recombination only occurs at <30% with 50 bp of homolog arm to the DFM1 gene. This can pose a challenge for labs that are new to yeast transformations as transformation efficiency can range widely across different laboratories.

### Potential solution

To improve targeted gene efficiency, the homology arm can be increased from 50 bp to 100 bp. Alternatively, an existing *dfm1Δ*-null strain can be used from the yeast knockout collection (available by Dharmacon) and the deletion KanMx cassette can be amplified with 0.5–1 kb of homology arm to the Dfm1 gene via amplification by PCR of the genomic data. This method has improved homologous recombination efficiency to >70%. Alternatively, any other strain with Dfm1 knocked out can be used as opposed to the yeast knockout collection.

### Problem 2

High background colonies are present in no DNA control transformation with antibiotic selection.

### Potential solution

Check if the yeast strain already contains the antibiotic resistance marker. For growth on antibiotic selection plates, it is possible that incorrect amount of antibiotic stock was added for making plates. Plates with the appropriate amount of antibiotics should be remade.

### Problem 3

No colonies on plates after yeast transformation.

### Potential solution

You can increase DNA amount to 2–3 mg per transformation, increase incubation time of competent cells with DNA, or remake PEG solution (barring the possibility that the solution was made incorrectly). For growth on antibiotic plates, it is suggested to increase recovery time incubation on YPD plates (up to 36 h) before being replica plated onto antibiotic selections plates.

### Problem 4

For non-induced conditions in raffinose, you still get basal levels of GAL promoter activity; increasing the tendency for *dfm1Δ*-null strains to suppress.

### Potential solution

For non-induced conditions, you can alternatively grow cultures in 2% glucose instead of 2% raffinose. In this case, glucose completely represses the GAL promoter. Prior to induction, rinse cells with sterile deionized water three times before transferring cells to minimal media supplemented with 1.8% raffinose/0.2% galactose.

### Problem 5

In the *in vivo* retrotranslocation assay, low lysing efficiency of cells can yield low overall western blot signal with anti-GFP and anti-ubiquitin.

### Potential solution

Lysis efficiency can be evaluated under the microscope using 20× magnification. Lysed cells are clearly distinguishable by their fragmented shape; for optimal yield it is critical to achieve ∼80%–90% lysis efficiency. If below this range, continue to vortex for up to three more 1-min cycles at 4°C. We use either 0.5 mm glass-based or silica-based beads for lysis (Biospec Products), but the lysis efficiency with silica-based beads appears to be somewhat higher.

### Problem 6

Ubiquitin signal is low in *in vivo* retrotranslocation assay.

### Potential solution

1) Ubiquitination is reversible and this modification can therefore easily be eliminated by deubiquitinases (DUBs). For this reason, it is essential to include DUB inhibitors such as NEM in the buffers used during the long incubation times used for immunoprecipitation in order to preserve the state of substrate ubiquitination (most DUBs are cysteine proteases that are inhibited by NEM).

2) For anti-ubiquitin blots, it is important to note that ubiquitin is small and difficult to denature and the ubiquitin epitopes might not be accessible to antibodies due to insufficient denaturation during SDS-PAGE or renaturation on the membrane. Therefore, after transfer to nitrocellulose membranes, the signal strength of anti-ubiquitin antibodies can frequently be enhanced significantly if the membrane is subjected to a denaturing treatment prior to blocking. Accordingly, the membranes are rinsed with water, sandwiched between sheets of Whatman paper, and placed in a glass dish. Deionized water is added to the dish and the membrane is boiled in a microwave oven at 3 × 1-min intervals; periodically check in between intervals to ensure that the water has not evaporated. This brings about a remarkable increase in signal strength, presumably due to revealing of cryptic epitopes from the heat

## Resource availability

### Lead contact

Further information and requests for resources and reagents should be directed to and will be fulfilled by the lead contact, Sonya Neal(seneal@ucsd.edu).

### Materials availability

Plasmids and yeast strains used in this study are available from our laboratory.

### Data and code availability

Original/source data for figures in the paper is available upon request. Original data have been deposited to Mendeley Data: https://doi.org/10.17632/ym9mtgmrwh.1.
